# Correlates of hepatic stiffness by FibroScan^©^ in a multicentric Italian cohort of HIV-infected patients

**DOI:** 10.1186/1758-2652-13-S4-P203

**Published:** 2010-11-08

**Authors:** P Tarquini, F Sozio, E Marchionni, E Gabrielli, E Polilli, F Ancarani, D Di Giammartino, LM Manzoli, G Parruti

**Affiliations:** 1 Teramo General Hospital, Unit of Infectious Diseases, Teramo, Italy; 2 Pescara General Hospital, Via Fonte Romana 8, Pescara, Italy; 3 Infectious Disease Clinics, University of Ancona, Unit of Hepatology, Ancona, Italy; 4 Pescara General Hospital, Unit of Clinical Pathology, Pescara, Italy; 5 University of Chieti, Epidemiology and Public Health Department, Chieti, Italy; 6 Pescara General Hospital, Unit of Infectious Diseases, Pescara, Italy

## Purpose of the study

The availability of FIBROSCAN© for non-invasive assessment of liver stiffness may be a valuable tool for investigating rates and correlates of liver fibrosis in HIV infected patients.

## Methods

Consecutive HIV patients followed at 3 Italian Institutions, with or without coinfection with hepatitis viruses, were enrolled Jan to Jun, 2010. Transient Elastography (FibroScan©) was performed by 1 physician per site, blinded to patients' data. Only procedures with ≥10 successful acquisitions and a success rate of ≥60% were evaluated. Elastographic results were expressed in KiloPascal (KPa, detection range 2.5 to 75).

## Summary of results

We included 214 patients, 146 (68.2%) males, mean age 44.5±9.1y (r. 22-79). As to risk factors, 154 (72.0%) were infected through heterosexual (61.0%) or homosexual (10.0%) exposure, the remaining due to drug abuse (26.6%) or blood transfusion (1.4%). Patients coinfected with Hepatitis C (69) or HCV/HBV (3) were overall 72 (33.8%), alcohol abusers 53 (24.8%), patients with a BMI ≥30 13 (6.0%). CD4 T-cell counts at the time of FIBROSCAN© were 509±275 (r. 23-1648), 185 (86.4%) patients being on HAART. ALT were ≥2UNL in 103 (48.1%) patients, normal or near-normal in the remaining patients. Mean platelet counts were 216±73 x103 (r. 56-429). Mean KPa values were 8.2±9.6 (r. 2.7-73). Univariate analyses revealed that higher liver stiffness scores were significantly associated with male gender (p=0.01), age (p=0.0004), coinfection with Hepatitis C/B (p<0.0001), parenteral transmission of HIV (p=0.0009), lower platelet counts (p<0.0001). They were near significantly associated with alcohol abuse (p=0.06) and higher BMI (p=0.13), not with being on HAART (p=0.5) and normal ALT values (p=0.6). Multivariate linear regression analyses revealed that only coinfection with Hepatitis C and/or B Viruses was independently associated with higher stiffness scores, whereas all other variables were not confirmed.

## Conclusions

Many variables have been reported as associated with increased liver stiffness in the HIV infected population. Our investigation reinforces that coinfection with hepatitis viruses plays an outstanding role in fostering liver fibrosis. Although considering other factors in HIV infected patients may be of value, curing hepatitis coinfections remains one major task to prevent end stage liver disease, even in a HIV population with a high prevalence of sexual transmission as ours.

